# MiR-129-5p alleviates depression and anxiety by increasing astrocyte ATP production partly through targeting deubiquitinase Mysm1

**DOI:** 10.1371/journal.pone.0322715

**Published:** 2025-05-09

**Authors:** Qiaozhen Qin, Heyang Zhang, Xiaotong Li, Huaqiang Ruan, Shuirong Liu, Yue Chen, Zhenhua Xu, Yan Wang, Xinlong Yan, Xiaoxia Jiang

**Affiliations:** 1 Beijing Institute of Basic Medical Sciences, Haidian, Beijing, P.R. China; 2 Beijing International Science and Technology Cooperation Base for Antiviral Drugs, Beijing Key Laboratory of Environmental and Viral Oncology, College of Chemistry and Life Science, Beijing University of Technology, Beijing, China; 3 Anhui Medical University, Hefei, Anhui, China; 4 Jishou University, Jishou, Hunan, China; University of Nebraska Medical Center College of Medicine, UNITED STATES OF AMERICA

## Abstract

Major depressive disorder (MDD) is a major global mental concern that severely affects quality of life, yet current pharmacological treatments remain limited in their effectiveness. Long-term chronic stress has been shown to increase the incidence of depression and anxiety. Micro RNAs (miRNAs) have been revealed to participate in the pathological process of depression and represent promising therapeutic targets. In this study, we found that microRNA-129-5p (miR-129-5p) was significantly decreased in the brains of depressive mice. Overexpression of miR-129-5p in the hippocampus effectively alleviated depressive-like behaviors and reduced the activation of microglial cells and astrocytes. In addition, ATP levels in depressive mice were significantly increased following miR-129-5p overexpression. The antidepressant effects of miR-129-5p were reversed when ATP function was blocked with the non-specific P2 receptor antagonist suramin. In vitro experiments revealed that miR-129-5p overexpression enhanced ATP production in astrocytes. Furthermore, using a dual-luciferase reporter assay, we found that miR-129-5p directly targeted Mysm1. When overexpressed in astrocytes, miR-129-5p significantly suppressed Mysm1 expression, promoted phosphorylation of p53 and AMPK, and enhanced the expression of PGC1α, factors previously associated with ATP production. Our findings highlight the crucial role of miR-129-5p in regulating depression, suggesting that miR-129-5p overexpression may serve as an effective strategy for antidepressant treatment.

## Introduction

Depression is a prevalent, costly, and debilitating condition, associated with an increased suicide of risk [1], it ranks as one of the leading global public health challenges. While currently available pharmacological treatments can be effective, they typically require up to 6 weeks to take effect, often prone to side-effects, and may require multiple recovery agents [2]. In today’s fast-paced society, young people encounter various forms of stress that can lead to mental health issues. Notably, the incidence of depression among young people, particularly females, has surged over the past decade [3]. This trend is particularly concerning given that adolescence is characterized by rapid social, emotional, and cognitive development along with key life transitions [4]. Although the exact pathogenesis of depression remains unknown, several potential causes have been recognized, including the monoamine hypothesis, alterations in the hypothalamic-pituitary-adrenal axis alterations, neuroinflammation, neuroplasticity, and epigenetic factors.

Noncoding RNAs (ncRNAs), including microRNAs (miRNAs), circular RNAs, and long ncRNAs, serve as crucial regulators of normal biological processes. Their dysregulation may contribute to the pathogenesis of various human diseases, including depression [5]. miRNAs are small, single-stranded ncRNAs that negatively regulate target gene expression by binding to partially complementary sequences in the 3′-untranslated region of their target mRNAs. Clinical studies have revealed various miRNA alterations presented in the cerebrospinal fluid, serum, and ventral prefrontal cortex of patients with major depressive disorder [6]. Isoliquiritin is one of the major flavonoid glycoside compounds extracted from Glycyrrhiza uralensis. It possesses a broad spectrum of pharmacological properties, including antiangiogenic, anti-neurotoxic, and anti-tumor [7]. Recent evidence has indicated that isoliquiritin could significantly alleviate depressive symptoms in mice by suppressing pyroptosis via the miRNA-27a/SYK/NF-κB axis and the NLRP3 cascade [8]. Additionally, Lilium Henryi Baker and Rehmannia Glutinosa Decoction (LBRD) is a traditional Chinese medicine formula that exhibits antidepressant activities by regulating miR-144-3p-mediated gamma-aminobutyric acid (*GABA)* synthesis and release [9]. Furthermore, emerging evidence indicates that miRNAs play essential roles in depression pathogenesis. Recent studies have shown that neurons secrete exosomes containing miR-9-5p to promote polarization of M1 microglia in depression [10]. In addition, microRNA-26a-3p rescues depression-like behaviors in male rats via preventing hippocampal neuronal anomalies [11]. Therefore, elucidating the mechanisms of miRNA regulation in depression neurogenesis may provide novel therapeutic strategies for individuals with depression.

Among the various types of ncRNAs, miRNAs have been extensively studied and identified as critical regulators of neural plasticity and higher brain functions [12]. miR-129-5p, a member of the miR-129 family, is widely expressed across tissues and organs in the human body, where it influences various biological processes [13]. Its dysregulation has been closely associated with the onset and progression of various malignant tumors, playing significant roles in either promoting or inhibiting tumor growth [14]. Additionally, miR-129-5p has emerged as a promising therapeutic target in Alzheimer’s disease, due to its crucial role in regulating the expression of target genes involved in Alzheimer’s disease pathogenesis [15].

Numerous studies have highlighted the involvement of abnormal neuronal function, hyperactive microglia, and heightened neuroinflammatory responses in the depression of development [16–18]. Recent evidence has implicated astrocyte dysfunction in the pathogenesis of major depressive disorder (MDD) [19]. Activated astrocytes release pro-inflammatory cytokines, such as IL-1β and TNFα, which are crucial in triggering depressive symptoms. As the most abundant glial cells in the brain, astrocytes are recognized for secreting ATP, which facilitates astrocyte-neuron communication. Several pathways for ATP secretion by astrocytes have been identified, with reduced ATP levels linked to depression-like conditions. Compromised ATP levels have been implicated in depressive disorders, and ATP replenishment is sufficient to modulate depressive-like behaviors in mice [20]. Under normal physiological conditions, extracellular ATP is primarily released by astrocytes. The calcium homeostasis modulator family protein (Calhm2) regulates astrocytic ATP release and neural activity. Conditional knockout of Calhm2 in astrocytes leads to reduced ATP concentrations and depressive behavior [21]. Additionally, hippocampal CD39 contributes to depression-like behavior induced by chronic social defeat stress through the hydrolysis of extracellular ATP [22]. The astrocytic release of ATP is triggered by increased intracellular calcium concentration [23], predominantly elicited via the inositol 1,4,5-trisphosphate (IP3) pathway[24]. Our previous study revealed that elevated Mysm1 in astrocytes exacerbated depressive-like behavior in mice induced by chronic stress and LPS. Suppression of Mysm1 expression could alleviate depressive disorders by promoting ATP production [25].

In this study, our screening revealed a notable reduction in miR-129-5p expression in the CRS depression model. We hypothesized that overexpression of miR-129-5p could ameliorate behavioral abnormalities in the mouse model by upregulating ATP related proteins.

## Materials and methods

### Animal

Eight-week-old male C57BL/6J mice were obtained from Beijing Vital River Laboratory Animal Technology (Beijing). The mice were housed in a controlled environment (23 ± 1 °C, 60 ± 5% humidity) with a 12-h light/12-h dark cycle (lights on at 07:00). Animals were allowed to habituate for 1 week prior to experimental procedures. Animals All animal experiments were conducted according to the Guide for the Care and Use of Laboratory Animals by the Administrative Panel on Laboratory Animal Care at the Institute of Basic Medical Sciences (Beijing, China). Mice were euthanized by cervical dislocation. The procedure was performed by firmly grasping the base of the tail with the dominant hand while stabilizing the mouse on a nonslip surface, followed by rapid traction of the tail with simultaneous cervical dislocation using the non-dominant hand at the neck region. This method resulted in immediate spinal cord separation and instantaneous death, as confirmed by cessation of vital signs.

### Chronic restraint stress (CRS) model

In this study, we utilized the chronic restraint stress (CRS) induced depression model. Mice were placed into a 50-mL conical tube (Falcon tube, Corning, Corning, NY, USA). The space between the mouse and the tube cap was filled with paper towels. The paper towels were designed to prevent mouse from turning freely in the conical tube. To prevent the mice from hypoxia, the conical tube was provided with air holes. The mice were restrained for six hours per day for 21 consecutive days [26]. The behavior tests were performed 24 h after CRS modeling.

### Lipopolysaccharide (LPS) model

The LPS modeling was performed according to the methods reported previously [17,27]. For the LPS-induced depression model, LPS (Sigma, L-2880) was dissolved in sterile 0.9% saline. Mice were exposed to LPS (0.5 mg.kg − 1 per day, intraperitoneal (i.p.)) for 7 days between 09:00 a.m. and 10:00 a.m. The behavior tests were performed 24 h after LPS modeling.

### Behavioral studies

Behavioral tests were performed by investigators who were blinded to study groups.

### Open field test (OFT)

The OFT was applied to evaluate anxiety and locomotor activity in rodents as previously described [28]. Animals were gently placed into open-field chambers (40 × 40 cm) which were equipped with video cameras. During the test, mice were allowed explore the arena for 5 minutes. Total distance traveled and time spent in the central area automatically were recorded.

### Elevated-plus maze test (EPMT)

The behavioral apparatus consisted of two open arms and two closed arms (width 5 cm × length 30 cm) elevated 50 cm above the floor and dimly illuminated [29]. Mice were placed individually in the center of the maze facing an open arm and allowed to freely explore for 5 minutes. The time spent or distance traveled in each arm were analyzed using a video tracking system. The maze was cleaned with 70% ethanol after each test to prevent olfactory influences from the previously tested mouse.

### Tail suspension test (TST)

The TST was performed as previously described with minor modifications [30]. A mouse with a medical tape placed 1 cm from the tip of the tail was suspended upside-down for 6 minutes on the TST instrument holder. The immobility time for each mouse throughout the last 4 minutes was statistically analyzed using ANY-MAZE software.

### Forced swimming test (FST)

The FST was performed as previously described with minor modifications [30,31]. Mice were placed in a transparent cylinder (diameter 10 cm, height 30 cm) containing 20 cm of water at 24 ± 1 °C. The total duration of immobility during the last 4 minutes of the 6-minutes session was analyzed.

### Stereotaxic injection

Stereotaxic injection was performed according to published work [32]. Mice were anesthetized with an intraperitoneal injection of 2,2,2-tribromoethanol (Sigma, 240 mg/kg of body weight). Standard surgery was performed to expose the brain surface above the hippocampus. Coordinates used for HIP injection were: bregma -1.70 mm, lateral ±1.25 mm, and dura -1.75 mm. The AAVs (HBAAV2/9-ZsGreen or HBAAV2/9-mmu-mir-129-1-ZsGreen) were stereotaxically injected with a glass pipette connected to a Nanoliter Injector 201 (World Precision Instruments, Inc.) at a slow flow rate of 0.15 ml/min to avoid potential damage to local brain tissue. AAV-ZsGreen only encodes GFP and does not include any non-targeting miRNA. The pipette was withdrawn at least 10 minutes after viral injection. Injections were bilateral. Behavioral tests were conducted 3 weeks after viral injection. Slice physiology and histology were conducted at least 3 weeks after AAV injection.

ATP (50 μM, 2 μl) or Suramin (5 μM, 2μl) were bilaterally microinjected into hippocampus according to the above guidelines.

### Cell culture and drug treatments

Mouse primary astrocytes were obtained from the hippocampus of 1-day-old neonatal C57BL/6J mice following a published protocol [33]. Briefly, hippocampal tissues were dissected and digested in 0.25% Trypsin-EDTA for 10 minutes at 37 °C, followed by mechanical shearing. After centrifugation (1000 rpm, 5 minutes), cells were resuspended in Dulbecco’s Modified Eagle Medium (DMEM/F12) supplemented with 10% fetal bovine serum (FBS). Then, the resulting suspension was filtered through a 70-μm filter and cultured in DMEM/F12 supplemented with 10% FBS, 40 U/mL penicillin and 40 μg/mL streptomycin. Medium was refreshed every 2–3 days. Upon reaching confluence (7–10 days), the cells were verified by GFAP immunostaining and were ready for use.

### Cell transfection

AAV-miR-129-5p and overexpression control (AAV-CON) were transfected using polybrene following the manufacturer’s protocols. Before transfection, cells were cultured in 6-well plates untill they reached 80% confluency. Then, astrocytes were added with AAV-miR-129-5p and AAV-CON for 48–72 hours to induce miR-129-5p overexpression. Adeno associated viruses (AAV-CON, AAV-miR-129-5p) were purchased from Hanbio Biotechnology Co., Ltd.

### Quantitative real-time polymerase chain reaction (qRT-PCR) analysis

Total RNA was extracted from the hippocampus and cells using TRIzol reagent (Invitrogen USA) and reverse transcribed using a first strand cDNA synthesis kit (Transgen Biotech, China) following the manufacturer’s guidelines. Actin was used as a normalization control for mRNA (Table 1). The sequences for the primers were as follows:

**Table 1:** Prime sequences.

Primer	Forward	Reverse
Actin	TCACTATTGGCAACGAGCGGTTC	CAGCACTGTGTTGGCATAGAGGTC
P2rx4	CTCATCCTGGCTTACGTCATT	GAATCCAAGCTGAGAAGTGTTG
P2ry2	GTCGTGGCTCTCTATATCTTCC	CGTAGTAATAAACCAACAGCGG
P2ry6	GCAAGGCGGCTCGTATGGC	TAGGCAGCAGCGAAGGTCTCC
GLAST	CAGAGAA GGTAAAATCGTGCAG	TTTAAAGCAGGCTTCTACCAGA
GS	TGAGAAAGTCCAAGCCATGTAT	CAGACTGAAAGGTACTAGAGCC
GLT-1	TTTTTGCTGGCATATTCCAAGC	AGATTATCTTCCAAGCAACGGA
Cx3cr1	AGCTCACGACTGCCTTCTTC	GTCCGGTTGTTCATGGAGTT
Aif1	CCGAGGAGACGTTCAGCTAC	GACCAGTTGGCCTCTTGTGT
Mysm1	AAGCACCGTTAGCCTCTTCGTTTC	CCTTCCGTCAGGACTCAGCAATG
CD68	GAAATGTCACAGTTCACACCAG	GGATCTTGGACTAGTAGCAGTG
TNFα	GGACTAGCCAGGAGGGAGAACAG	GGACTAGCCAGGAGGGAGAACAG
IL6	TCTGGAGCCCACCAAGAACGATAG	GTCACCAGCATCAGTCCCAAGAAG

### Luciferase assays

The potential miR-129-5p binding sites in the Sclerostin 3′UTR were predicted using TargetScan (http://www.targetscan.org) [34]. The luciferase reporter assay was performed as previously reported. The relationship between miR-129-5p and Mysm1 was confirmed by luciferase reporter assay. The sequences containing the wild-type (Mysm1-wt) or mutant (Mysm1-mut) seed region of Sclerostin were synthesized and cloned into a luciferase reporter plasmid. The host 293T cells were seeded in 48-well plates at a density of 1 × 10^4 cells per well and transfected with the indicated reporter construct and a Renilla luciferase plasmid. Twenty-four hours after transfection, the activities of firefly and Renilla luciferase were measured using a fluorescence spectrophotometer (Thermo Multiskan FC) according to the manufacturer’s instructions. The relative transcriptional activity was normalized to the corresponding vehicle control value.

### Western blot

The Western blot was performed following a published procedure with minor modifications [35]. Proteins were extracted from the hippocampus of CRS mice and from treated astrocytes, and total protein concentrations were measured using the Bradford assay kit. Proteins were separated on 12% SDS-PAGE gels and then transferred to polyvinylidene difluoride (PVDF) membranes. After blocking with 5% skim milk, the membranes were incubated with primary antibodies at 4 °C overnight. Then, appropriate HRP-conjugated secondary antibodies were added and incubated for 1.5 hours at room temperature. The blots were visualized using ECL western blotting detection reagents. The membranes was probed at 4 °C overnight with the following primary antibodies: Mouse anti-GFAP (1:1000, Millipore, 3380386), Rabbit anti-MYSM1 (1:1000, Abcam, ab190381), Rabbit anti-PGC1α (1:1000, Cell Signaling Technology, 2187),Rabbit anti-p-p53 (1:1000, Cell Signaling Technology, 9284), Rabbit anti-p53 (1:1000, Cell Signaling Technology, 2524), Rabbit anti-p-AMPK (1:1000, Cell Signaling Technology, 2535), Rabbit anti-AMPK (1:1000, Cell Signaling Technology, 2532), Rabbit anti-Sirt1 (1:1000, Cell Signaling Technology, 8469), Mouse anti-β-actin (1:1000, APPLYGEN, C1313-100 or Cell Signaling Technology, 4907). After three washes, the membranes were incubated with secondary antibodies in TBS buffer for 1 hours at 22–24 °C. Enhanced chemiluminescence was used to visualize the immunoreactive bands. The secondary antibodies included HRP goat anti-rabbit IgG (1:2000, Abclonal, AS014) and HRP goat anti-mouse IgG (1:2000, Abclonal, AS003).

### Immunofluorescence

The expression of the primary antibody was determined by immunocytochemistry as previously described [36]. Mouse brains were dissected and fixed with 4% paraformaldehyde in PBS for 24 hours at room temperature. Then, the brains were dehydrated with a sucrose gradient (15% and 30%) in PBS. Coronal brain slices (40 μm) were sectioned using a Leica CM3050S cryostat (Leica). Brain slices were washed in PBS twice and incubated in blocking buffer (PBS containing 0.3% Triton X-100, 10% donkey serum) for 1 hours at room temperature. The primary antibodies used were GFAP (Millipore, MAB360), Iba1 (Abcam, ab178847) and Mysm1 (Abcam, ab193081). The slices were incubated with fluorescence-conjugated secondary antibodies (Jackson Immunoresearch, Donkey anti-rabbit IgG H&L (Cy3, 1: 200) or Donkey anti-mouse IgG H&L (Alexa Fluor® 488, 1: 200)) for 1 hours at room temperature. Images of stained brain slices were acquired using a confocal microscope (Leica).

### Immunofluorescent intensity analysis

Randomly selected high-power Fields corresponding to the peri-impact and hippocampus regions were used to count positive cells in this region [37]. Briefly, 30-μm cryostat-sectioned tissues were examined at 40 × magnification. Analysis of the mean fluorescence intensity (MFI) for confocal images was conducted using ImageJ software. From all sections, approximately 4–5 images were taken from each coronal section using confocal microscopy (Olympus). All photomicrographs were converted to grayscale. Background was selected from blank control images, and subsequently used to subtract the background from all images. All tissue sections were stained in one batch, with the same imaging threshold and exposure time to ensure consistency for image analysis. Thereafter, the staining intensity of each section was quantified as the average optical density readings of four randomly selected areas within that section. The final staining intensity for each group was calculated as the average of the staining intensity per section.

### ATP detection

Fresh brain tissue was dissociated within 5 minutes. ATP levels were measured using an enhanced ATP assay kit (S0027 Beyotime Biotechnology, Shanghai, China) according to the manufacturer’s instructions. In brief, the collected samples were rapidly frozen in liquid nitrogen for 30 seconds and homogenized in lysis buffer for 1 minute on ice. The lysates were then collected and centrifuged at 12,000 × g for 5 minutes at 4 °C. In a 96-well plate, 20 μL supernatant was added to the wells containing 100 μL of ATP assay working dilution. Luminescence was detected using a multifunctional microplate reader (Infinite M200 PRO; Tecan, Switzerland). The protein concentration of each group was determined and used to calibrate the cellular ATP levels. Finally, the ATP concentration was calculated using the derived ATP standard curve and normalized to the protein concentration of the supernatants.

### Statistical analysis

Prior to analyses, we confirmed data normality using D’Agostino-Pearson and homogeneity of variances using Barlett’s test. Differences among groups were analyzed by one-way analysis of variance (ANOVA), followed by the Tukey’s multiple-comparison tests. All data sets followed a normal distribution and were presented as the mean ± standard error of the mean (SEM). For comparison between two groups, data were analyzed using unpaired Student’s t test. For multiple group comparison, data were analyzed using one-way or two-way ANOVA with Tukey post hoc test. The criterion for statistical significance was set at p < 0.05.

## Results

### MiR-129-5p levels were decreased in depression mouse models

**Fig 1:**
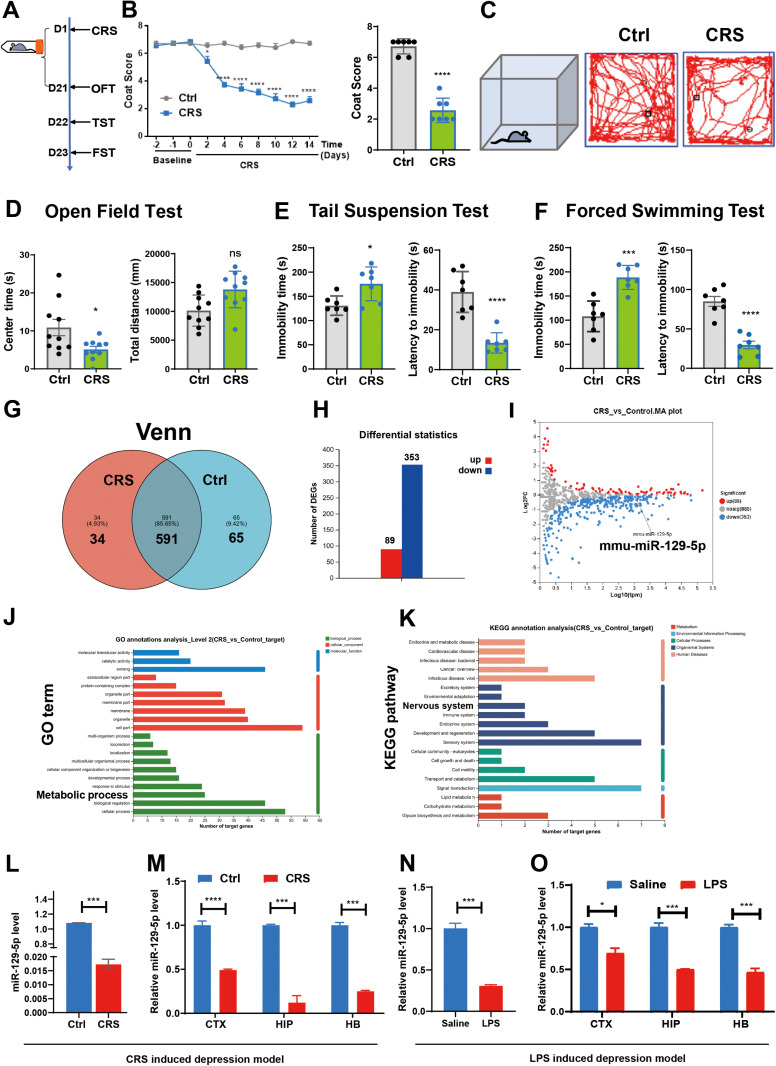
MiR-129-5p levels were decreased in mouse depression models. **A.** Experimental schedule. All mice underwent behavioral tests following CRS treatment. **B.** Coat scores were assessed for the control and CRS groups from 0 to day 14. **C.** Representative route maps were conducted in the open field tests. **D.** Center time and total distance traveled by the control and CRS groups were displayed in the open field tests. **E, F.** Immobility time (left) and latency to immobility time (right) were recorded for the control and CRS groups in the tail suspension test (E) and in the forced swimming test **(F)**. **G.** A Venn diagram illustrated differentially expressed miRNAs between control and CRS mice. **H.** Histogram depicting the distribution of increased and decreased miRNAs between control and CRS mice. **I.** Volcano plot illustrated the differentially expressed miRNAs between control and CRS mice. The Y-axis represents the fold change, while the X-axis indicates the significance of differential expression. The gray points represent miRNAs with no significant change (p < 0.05, false discovery rate (FDR) q < 0.05), while red and blue points indicate upregulated and downregulated miRNAs (p < 0.05, FDR q < 0.05), respectively. **J.** Gene ontology enrichment analysis quantifies target genes in each term. The richness factor was calculated by dividing the number of target genes by the total number of genes within each term. The scatterplot indicates the number of GO target genes, p-value and richness factor. **K.** The KEGG pathway analysis identifies the number of target genes within each pathway. Those with a p-value < 0.05 are considered significant. **L.** Relative expression of miR-129-5p in the control and CRS groups. **M.** Relative expression of miR-129-5p in the CTX, HIP, and HB of control and CRS mice. **N.** Relative expression of miR-129-5p in the saline and LPS-treated group. **O.** Relative expression of miR-129-5p in the CTX, HIP, and HB regions of saline and LPS treated group. (n = 3 - 10 mice per group; Data are presented as the mean ± standard error; *, **, ***, and **** indicate significance at p < 0.05, p < 0.01, p < 0.001, and p < 0.0001, respectively.).

In this study, male C57BL/6 mice were subjected to 3 weeks of CRS to establish a depression model. We conducted multiple behavioral tests to assess depressive behaviors (Fig 1A). As expected, the CRS model exhibited depressive-like phenotypes, evidenced by coat score assessments over 14 days that showed progressive hair deterioration characteristic of depression (Fig 1B). We observed significant differences in the time spent in the center of the arena during the OFT between the groups (Figs 1C and 1D), along with prolonged immobility times in the TST and FST for the CRS group compared to the control (Figs 1E and 1F). Additionally, CRS-treated mice displayed reduced latency to immobility, providing further validation of the depression model (Figs 1E and 1F).

We further conducted small RNA sequencing to identify differentially expressed miRNAs between control (Ctrl) and CRS hippocampus tissues. Our analysis revealed 353 miRNAs with decreased expression in CRS mice (Figs 1G, and 1H). Interestingly, recent research has found that in the brain-derived extracellular vesicle (EVs) of patients with depression, the expression of specific miRNAs, such as miR-129-5p, is reduced [38]. Among these, miR-129-5p displayed a fold change difference and significant differential expression in at least one region (Fig. 1I).

Energy metabolism refers to the processes of energy acquisition, transformation, and utilization within living organisms, with ATP (adenosine triphosphate) playing a central role in this process [39]. ATP is the primary source of cellular energy and plays a crucial role in modulating depressive-like behaviors in adult mice [40]. Moreover, the levels of ATP in the brains of mice with depression are reduced [40]. Gene Ontology analysis and KEGG analysis also revealed enrichment for functions related to metabolic processes (Fig 1J) and nervous system development (Fig 1K). The expression differences were validated through qRT-PCR, specifically examining the CTX, HIP and HB regions of CRS mice following 3-week exposure period. Both CRS exposure and LPS induced depressive-like behaviors resulted a notable reduction in miR-129-5p expression levels (Figs 1L-1O).

MiR-129-5p has been extensively studied for its diverse cellular functions, notably its role in mediating LINC00574 and reducing UGT2B15 expression in HepaRG cells [41]. Moreover, elevated level of hsa-miR-129-5p has been observed in scar tissues, where it significantly influences cellular processes including growth, proliferation, differentiation, adhesion, migration and apoptosis. Hsa-miR-129-5p also plays a crucial role in regulating wound healing in thermally injured human epidermal stem cells [42]. However, the specific involvement of mus-miR-129-5p in CRS-induced depression remains unexplored, presenting a potential direction for future research investigations.

### Overexpression of miR-129-5p alleviated depression like behaviors

Adeno-associated virus (AAVs)-mediated miR-129-5p overexpression was established in CRS mice to assess its potential in alleviating depressive phenotypes *in vivo*. Male C57/BL6J mice were subjected to 3 weeks of CRS, following by bilateral hippocampal microinjections of AAV-miR-129-5p or AAV-CON (GFP only), as detailed described in the method section.

**Fig 2:**
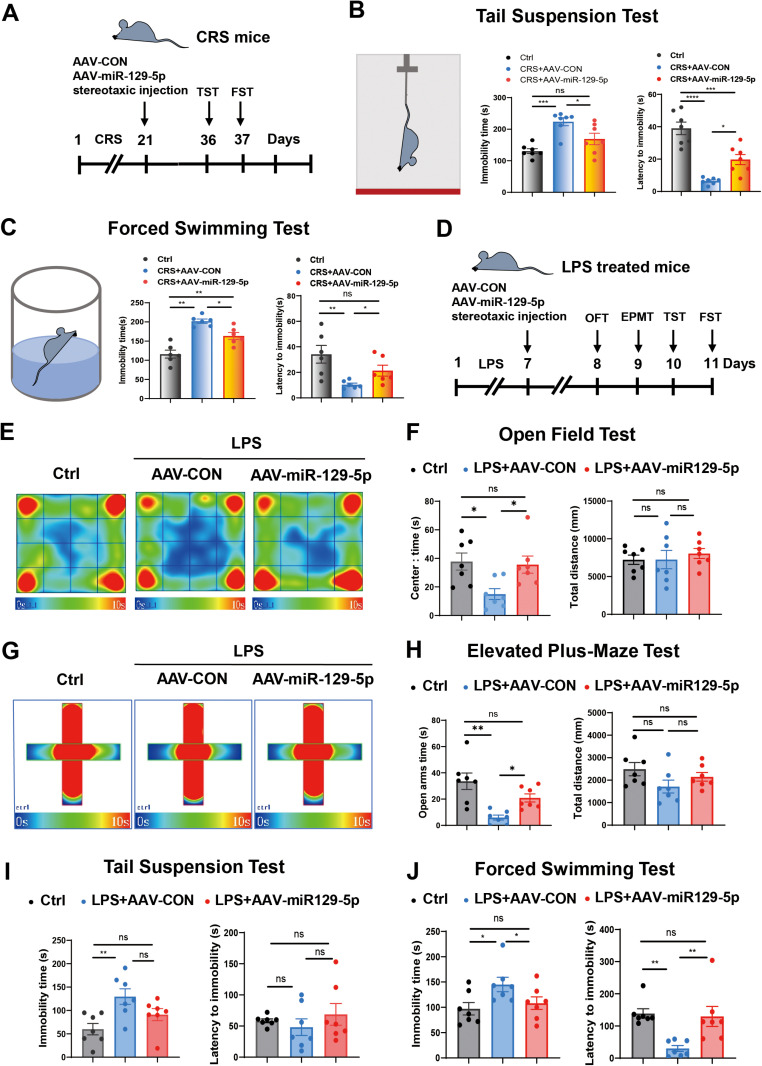
Overexpression of miR-129-5p alleviated the depressive-like phenotypes of CRS and LPS treated mice. **A.** Experimental schedule. The experimental paradigm involved virus injection into depressive mice following CRS treatment. **B, C.** Immobility time and latency to immobility of each group in the tail suspension test (B) and in the forced swimming test **(C)**. **D.** Experimental schedule. The experimental paradigm illustrated the virus injection into depressive mice following LPS treatment. **E.** Typical route map of control, AAV-CON, and AAV-miR-129-5p LPS-treated mice were displayed in open field test. **F.** Center time duration and total distance traveled by each group were displayed in the open field test. **G.** Heatmap of control, AAV-CON, and AAV-miR-129-5p of LPS treated mice in the elevated plus-maze test. **H.** Time spent in open arms and total distance traveled by the three groups were measured in elevated plus-maze test. **I, J**. Immobility time and latency to immobility of each group in the tail suspension test (I) and in the forced swimming test **(J)**. (n = 6-7 mice per group; Data are presented as the mean ± standard error; *, **, ***, and **** indicate significance at p < 0.05, p < 0.01, p < 0.001, and p < 0.0001, respectively.).

CRS-induced depressive-like phenotypes were significantly alleviated in mice following AAV-miR-129-5p administration, as demonstrated in the TST and FST assays (Fig 2A). Specifically, significant reductions in immobility durations were observed in mice injected with AAV-miR-129-5p microinjection in the HIP compared to the controls (Figs 2B and 2C).

Fig 2D illustrated the experimental paradigm for treating LPS induced depressive mice. Behavioral tests were conducted to evaluate the effects of miR-129-5p on anxiety and depression-like behaviors. In the open field test, overexpression of miR-129-5p significantly reduced anxiety in LPS-treated mice, as evidenced by the increased center time in mice injected with AAV-miR-129-5p, compared to AAV-Con (Figs 2E and 2F). The elevated plus-maze test results showed that miR-129-5p overexpression decreased depression-like behavior, evidenced by increased time in open arms for AAV-miR-129-5p compared to AAV-CON (Figs 2G and 2H). LPS-treated mice with miR-129-5p overexpression in the hippocampus exhibited significantly shorter immobility times in both the TST and FST assays, indicating reduced depressive-like behavior (Figs 2I and 2J). These findings indicate that elevating miR-129-5p levels in the hippocampus can effectively alleviate depression and anxiety behaviors, highlighting its potential therapeutic value.

### Overexpression of miR-129-5p reduced microglia activation in depression mouse models

Microglia, the resident immune cells in the brain, play a crucial role in modulating neuroinflammation and depression [43]. As the primary responders in the innate immune system of the central nervous system, these cells adaptively regulate the microenvironment in both healthy and pathological states, influencing inflammation, synaptic refinement, pruning, and neuronal connectivity [44]. Consequently, depression has been increasingly recognized as a disorder linked to microglial activity, a condition often referred to as “microgliopathy” [45].

**Fig 3:**
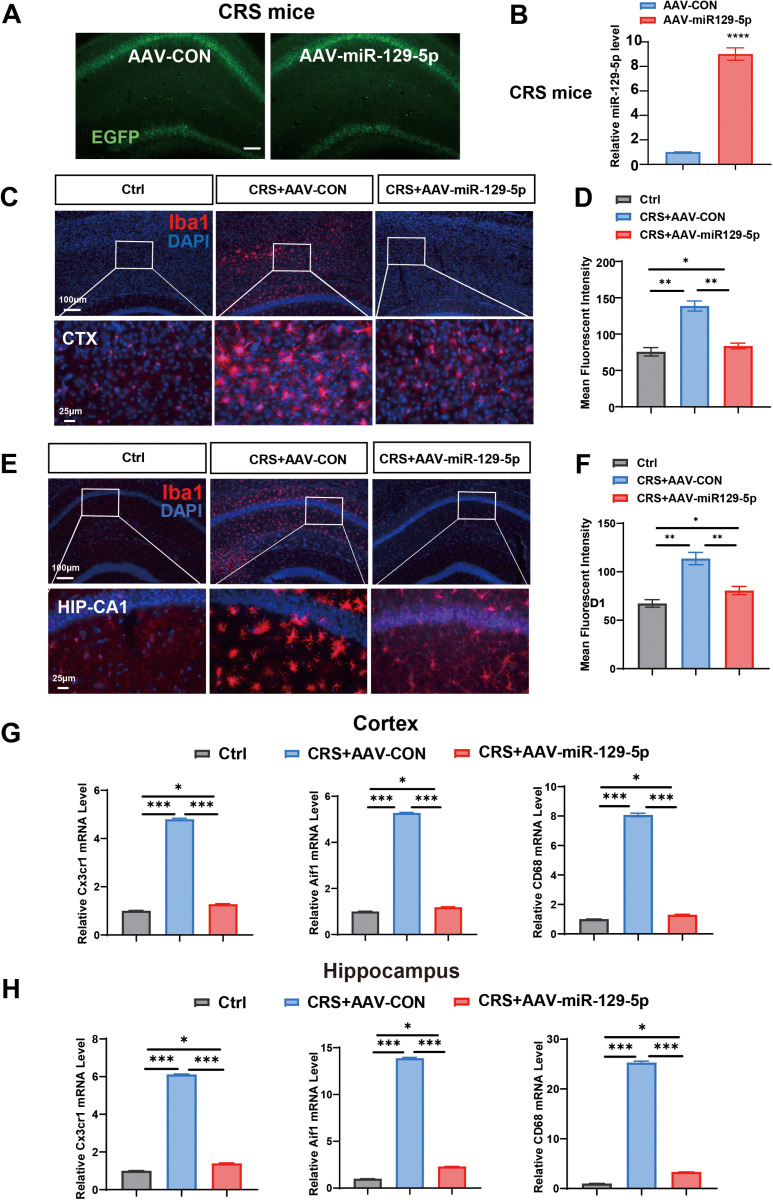
Stereotactic injection of AAV-miR-129-5p attenuated microglia activation in depressed mice. **A.** EGFP fluorescence visualization in brain tissues three weeks after the stereotactic injection of AAV-CON and AAV-miR-129-5p virus. Scale bar, 100 μm. **B.** miR-129-5p expression was assessed by qRT-PCR analysis in the brain tissues. **C.** Representative Iba1-stained coronal sections of murine brain cortex from each group. Scale bar, 100 μm and 25 μm. **D.** Quantification of the mean fluorescence intensity of Iba1 in the cortex. **E.** Representative Iba1-stained coronal sections of murine hippocampus from the three groups. Scale bar, 100 μm and 25 μm. **F.** Quantitative analysis of the mean fluorescence intensity of Iba1 in the hippocampus. **G, H** The expression of Cx3cr1, Aif1 and CD68 was assessed by qRT-PCR in the cortex (G) and hippocampus of each group **(H)**. (n = 3 - 4 mice per group; Data are presented as the mean ± standard error; *, **, ***, and **** indicate significance at p < 0.05, p < 0.01, p < 0.001, and p < 0.0001, respectively.).

The EGFP fluorescence post-AAV injection is depicted in Fig 3A, indicating successful virus introduction. Furthermore, miR-129-5p levels were elevated in the AAV-miR-129-5p injection group (Fig 3B). Microglial activation was assessed using Iba1 labeling, where activated microglia (Iba1-positive) were characterized by enlarged cell bodies and axonal coarseness. Notably, Iba1 labeling intensity significantly increased in the cortex and hippocampus of CRS mice, indicating enhanced microglial activation (Figs 3C and 3E). However, the AAV-miR-129-5p injection markedly attenuated this activation, as observed in the intensity quantification data (Figs 3D and 3F). Analysis of microglial markers (Cx3cr1, Aif1, and CD68) revealed elevated expression levels in the CRS group, but significantly reduced level in the CRS mice treated with AAV-miR-129-5p, compared to the Ctrl group (Figs 3G and 3H). These findings suggest that miR-129-5p can effectively modulate microglial activation.

### Overexpression of miR-129-5p decreased astrocytes activation in depression mouse models

Astrocytes, the predominant type of glial cells, play crucial roles in maintaining brain health by regulating ion and neurotransmitter levels, supporting neural and synapse development, and enhancing neuronal metabolism [46]. However, under certain conditions, astrocytes undergo reactive transformations, during which they lose their supportive functions and begin secreting pro-inflammatory cytokines, chemokines, and growth factors, ultimately contributing to increased brain inflammation.

**Fig 4:**
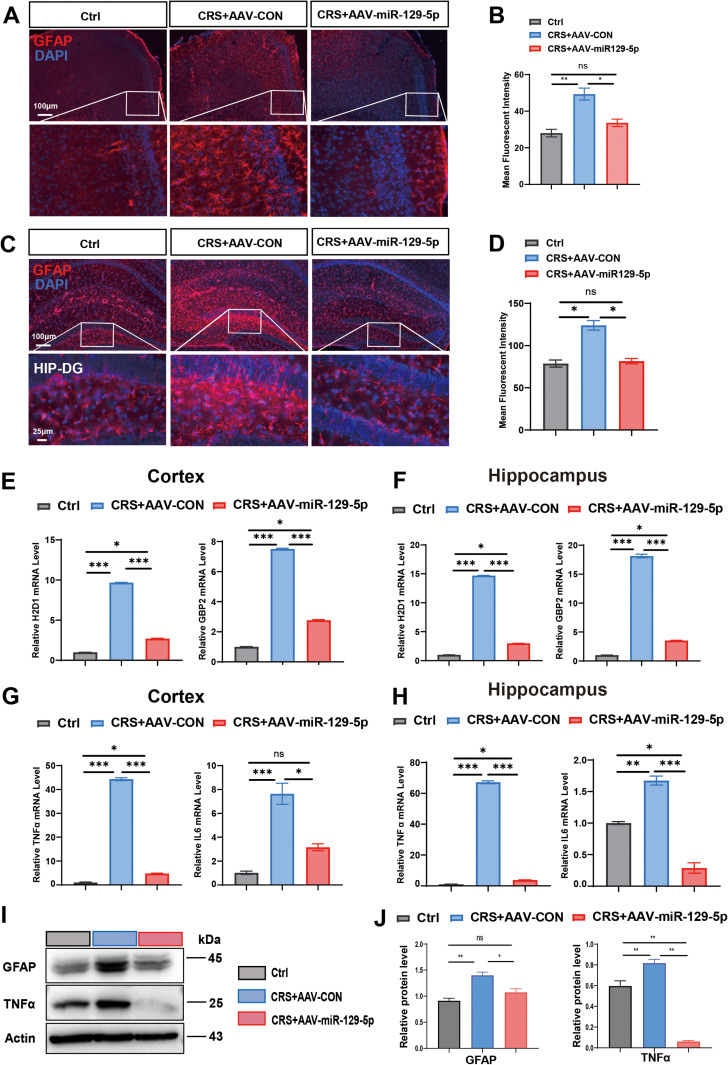
Stereotactic injection of AAV-miR-129-5p suppressed astrocyte activation in depressed mice. **A.** Representative GFAP-stained coronal sections of murine brains from the three groups in the cortex. Scale bar, 100 μm and 25 μm. **B.** Quantification of mean fluorescent intensity of GFAP in each group within the cortex. **C.** Representative GFAP-stained coronal sections of murine hippocampus from the three groups. Scale bar, 100 μm and 25 μm. **D.** Quantification of mean fluorescent intensity of GFAP in each group in the hippocampus. **E, F.** Expression levels of H2D1 and GBP2 in the cortex (E) and in the hippocampus (F) of each group, as detected by qRT-PCR. **G, H.** Expression levels of the pro-inflammatory factors TNFα and IL6 in the cortex (G) and hippocampus (H) of the three groups, as detected by qRT-PCR. **I.** Western blot analysis of GFAP and TNFα in the cortex and hippocampus following injection with control (AAV-CON) and miR-129-5p overexpression virus (AAV-miR-129-5p), respectively. **J**. Relative protein expression levels of GFAP and TNFα in the three groups. (n = 3 - 4 mice per group; Data are presented as the mean ± standard error; *, **, ***, and **** indicate significance at p < 0.05, p < 0.01, p < 0.001, and p < 0.0001, respectively).

Astrocytes were identified using glial fibrillary acidic protein (GFAP) labeling in brain tissues across four experimental groups (Figs 4A and 4C). GFAP immunofluorescence analysis revealed that astrocytes in CRS mice exhibited increased sizes and more extensive branching. The GFAP labeling intensity for the AAV-miR-129-5p-injected CRS mice was similar to that of the Ctrl group, but significantly reduced relative to the CRS-only groups (Figs 4B and 4D). Astrocyte activation was assessed using qRT-PCR, which showed significant reductions in the A1 astrocyte-specific transcripts H2D1 and GBP2 in the AAV-miR-129-5p treated CRS mice (Figs 4E and 4F). Similarly, the pro-inflammatory markers TNFα and IL6 expression were also notably decreased in the AAV-miR-129-5p-treated CRS group compared to the CRS group (Figs 4G and 4H). Western blot analysis further confirmed the reductions of both GFAP and TNFα in the CRS mice following AAV-miR-129-5p administration (Figs 4I and 4J). Among them, the level of TNFα was lower than that of the normal group after treatment with miR-129-5p, which may be due to the over-suppression of the inflammatory response by AAV-miR-129-5p.These findings suggest that AAV-miR-129-5p administration effectively attenuates astrocyte activation in CRS mice.

### Overexpression of miR-129-5 relieved depression-like behavior by increasing ATP production

**Fig 5:**
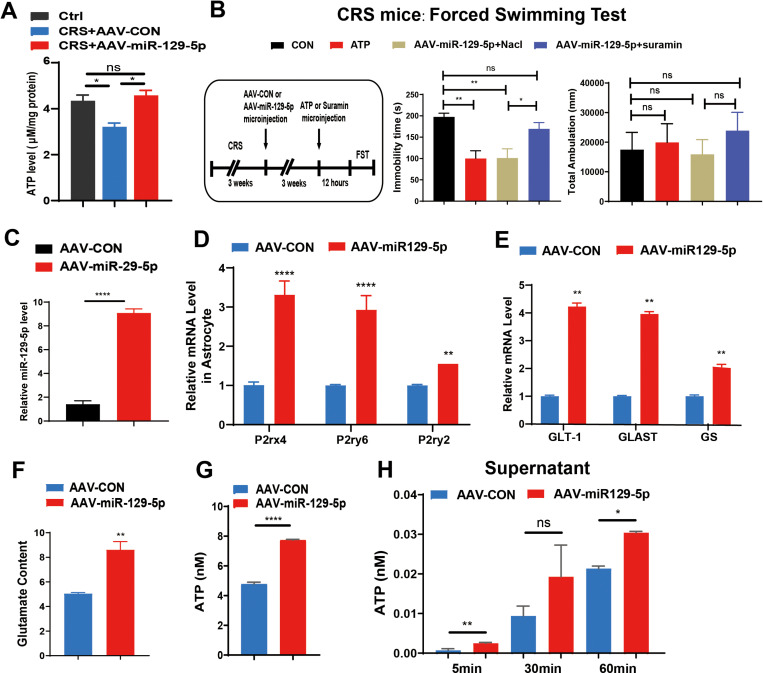
Overexpression of miR-129-5p alleviated depression-like behaviors by increasing ATP content. **A.** Relative miR-129-5p levels in brain tissues of the three groups. **B.** Immobility time (left) and latency to immobility time (right) of each group in the forced swimming test. **C.** Relative expression levels of miR-129-5p in primary astrocytes following virus infection. **D.** Relative expression levels of P2rx4, P2ry2, and P2ry6 in primary astrocytes. **E.** Relative expression levels of GLT-1, GLAST, and GS in the two groups. **F.** Comparison of glutamate content between the control (AAV-CON) and miR-129-5p overexpression (AAV-miR-129-5p) groups. **G.** ATP levels measured in the supernatant of primary astrocytes after transduction with the indicated control virus (AAV-CON) or miR-129-5p overexpression virus (AAV-miR-129-5p). **H.** ATP levels measured in primary astrocyte supernatant after transduction with the indicated control virus (AAV-CON) or miR-129-5p overexpression virus (AAV-miR-129-5p). (n = 3 - 10 mice per group; Data are presented as the mean ± standard error; *, **, ***, and **** indicate significance at p < 0.05, p < 0.01, p < 0.001, and p < 0.0001, respectively).

Given the critical role of astrocytes in neuroinflammation and brain energy metabolism, we investigated whether miR-129-5p modulates astrocyte function and ATP levels [47–49]. We assessed ATP content in the cortex and hippocampus and found that AAV-miR-129-5p injections could increase ATP concentration (Fig 5A). Subsequently, we administered ATP, AAV-miR-129-5p, and NaCI or suramin into mice to determine whether ATP content was a determining factor. The results demonstrated that both ATP or AAV-miR-129-5p injections significantly reduced the immobility time in mice (Fig 5B).

Additionally, we isolated primary astrocytes and transfected them with AAV-miR-129-5p virus, resulting a significant increase miR-129-5p levels in astrocytes (Fig 5C). The P2 purinergic receptor family, which is implicated in numerous physiological processes, such as neurotransmission, mechanical adaptation, and inflammation, consists of ATP-gated non-specific cation channels (P2XRs) and G-protein coupled receptors (P2YRs). Subsequently, using qRT-PCR, we detected significantly increased mRNA levels of ATP receptors P2rx4, P2ry2, and P2ry6 (Fig 5D).

Glutamate, the principal excitatory neurotransmitter in the central nervous system (CNS), plays a crucial role in rapid signal transmission, learning, memory, and synaptic plasticity. To prevent excitotoxic neuronal death caused by elevated levels of extracellular glutamate, astrocytic glutamate transporters remove glutamate from the synapse following impulse transmission, thereby maintaining optimal glutamate concentrations [50,51]. Additionally, we observed increased mRNA expression of glutamate transporters and enhanced glutamate phagocytosis (Figs 5E and 5F). Using an ATP detection kit, we assessed ATP content in both astrocytes and the culture medium supernatant, which revealed a significant increase (Figs 5G and 5H).

As demonstrated, Overexpression of miR-129-5p alleviated depression-like behaviors by enhancing ATP receptor expression and increasing ATP content.

### Mysm1 is a direct target of miR-129-5p

**Fig 6:**
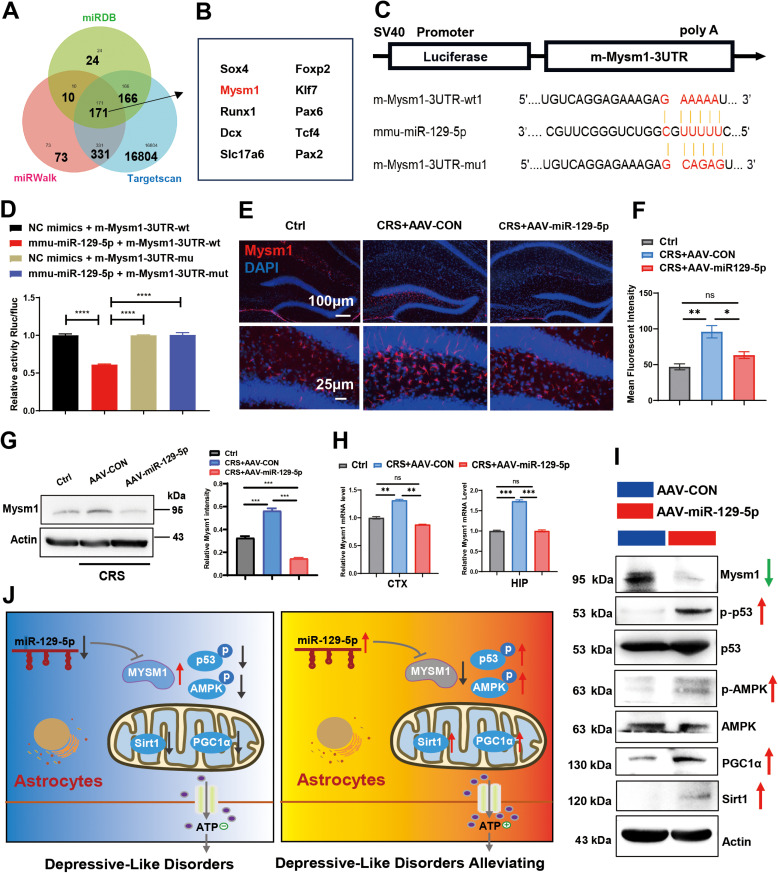
Relationship between miR-129-5p and Mysm1 in depressed mice. **A.** A Venn diagram displaying potential targets of miR-129-5p analyzed by TargetScan, miRDB, and miRWalk analyses. **B**. The Mysm1 gene was identified and chosen for further in-depth study. **C.** Bioinformatics analysis revealed the prediction of miR-129-5p binding sites within the 3′UTR of Mysm1 mRNA. **D.** Luciferase activity assays of Mysm1 3′UTR reporters in 293T cells transduced with miR-129-5p, miR-129-5p-mutant, or negative control. The relative luciferase activity in negative control-transfected 293T cells was designated as 1. **E.** Representative images of immunofluorescence staining with Mysm1 (red) antibody and DAPI (blue) in the hippocampus. Scale bar, 100 μm and 25 μm. **F.** Quantification of mean fluorescent intensity of Mysm1 in the hippocampus region. **G.** Protein expression levels of Mysm1 were examined by western blotting assays (left) and quantification of Mysm1 was shown (right). **H.** The expression levels of Mysm1 were analyzed by qRT-PCR. **I.** Western blot analysis was performed to show the the protein levels. **J.** Schematic diagram illustrates the miR-129-5p/Mysm1 axis in astrocytes during depression. After injecting AAV-miR129-5p into depressive mice, Mysm1 expression was downregulated in the astrocytes. Inhibition of astrocytic Mysm1 alleviates depressive-like disorders by activating the p53 and AMPK pathways, thus enhancing ATP production. (n = 3 - 4 mice per group; Data are presented as the mean ± standard error; *, **, ***, and **** indicate significance at p < 0.05, p < 0.01, p < 0.001, and p < 0.0001, respectively).

To identify the downstream genes regulated by miR-129-5p, we utilized three prediction tools (TargetScan, miRDB, and miRWalk) to explore potential targets (Fig 6A). Among the predicted targets, we specifically focused on Mysm1 (Fig 6B), which emerged as a promising candidate due to its role in the p53 signaling pathway and potential relevance to ATP production. The binding sites of miR-129-5p within the 3′ UTR of Mysm1 mRNA were illustrated (Fig 6C). We further utilized a luciferase reporter assay to validate the direct interaction between miR-129-5p and Mysm1. Following the transfection of constructs into 293T cells, luciferase activity was significantly reduced in cells co-transfected with wild-type Mysm1 3′ UTR and miR-129-5p at 36 hours (0.60-fold, p < 0.01) (Fig 6D). In vivo studies, the AAV-miR-129-5p treated CRS mice exhibited lower Mysm1 levels compared to the untreated CRS group (Figs 6E and 6F). Western blot analysis further confirmed the decreased Mysm1 expression in the brains of CRS mice overexpressing AAV-miR-129-5p (Fig 6G). These findings were corroborated by qRT-PCR analysis (Figs 6H). Following transfection of primary astrocytes with AAV-miR-129-5p, western blotting analysis demonstrated significant reductions in Mysm1 levels, along with increased phosphorylation of both p53 and AMPK (Fig 6I).

## Discussion

In this study, we investigated the antidepressant effects of miR-129-5p and its underlying mechanisms. Our findings demonstrate that miR-129-5p effectively alleviates depressive symptoms and anxiety-related behaviors induced by either CRS or LPS. Treatment with AAV-miR-129-5p reduced both astrocyte and microglia activation, along with increased astrocyte ATP production. Fig 6J illustrates the schematic representation of the miR-129-5p/Mysm1 axis in the astrocytes of depressive mice. The interaction between miR-129-5p and the Mysm1/p53/AMPK/PGC1α/Sirt1 pathway provides novel insights into depression research and potential therapeutic interventions.

The CRS paradigm has been extensively used to explore underlying mechanisms of depression- and anxiety-related behaviors [52]. In real-life situations, individuals frequently encounter stressors arising from social interactions with others, and social challenges appear to be among the most prevalent stressors affecting both humans and social animals. When rodents are repeatedly exposed to restraint stress, they demonstrate significant symptoms, including anhedonia, behavioral despair, social avoidance, and elevated anxiety levels. The LPS depression model is widely used to investigate inflammation-associated depression. LPS, a component of the cell membrane of Gram-negative bacteria, triggers the production of pro-inflammatory cytokines in both the brain and periphery [53]. In rodents, LPS administration induces sickness behaviors, characterized by decreased body weight, food intake, and locomotor activity, which typically resolve after 14–18 hours. Subsequently, mice enter a phase of depression-like behaviors characterized by declined sucrose preference rate and prolonged immobility duration in both the TST and FST assays [54,55]. In our study, mice subjected to either CRS or LPS exhibited prolonged immobility in the TST and FST, suggesting the successful development of depression-like behavior following these experimental challenges.

In a previous study, miR-129-5p was shown to alleviate behavioral despair in mice with post-stroke depression [56]. Recent research revealed that exosome derived from human umbilical cord mesenchymal stem cells (hucMSC-Ex) attenuate inflammatory bowel disease (IBD) by miR-129-5p targeting of ACSL4, which inhibits lipid peroxidation (LPO) and ferroptosis, thereby reducing intestinal inflammation and repairing damage [57]. In our study, the administration of AAV-miR-129-5p significantly improved the behavior performance in TST, FST, and OFT tests, indicating that miR-129-5p effectively alleviated depression in mice. We further demonstrated that miR-129-5p exhibits potent antidepressant-like activity in the rodent depression models. Additionally, a previous study has reported the antidepressant effects of miR-129-5p in the chronic unpredictable mild stress (CUMS) model [58]. Our results further confirm the universality of miR-129-5p in treating depression and highlight its significant potential as an antidepressant target. Importantly, beyond its anti-inflammatory effects, we found that miR-129-5p targets Mysm1 to regulate astrocyte metabolism. This discovery further elucidates the multiple mechanisms underlying the antidepressant effects of miR-129-5p and underscores its significance for further research.

Neuroinflammation, characterized by an abnormal immune response in the brain, has gained increasing attention for its association with MDD. Persistent neuroinflammation could trigger depressive-like behaviors, creating a complex feedback loop [59]. Both microglia and astrocytes play a crucial role in regulating inflammation, synaptic plasticity, and neural network formation, which are key factors affecting depression. In this study, we specifically highlighted the involvement of microglia and astrocytes in the pathology of depression. We found that overexpression of miR-129-5p effectively reduced the activation of microglia and astrocytes in depression mouse models. However, the precise mechanism by which miR-129-5p inhibits the activation of astrocytes and microglia requires further study.

Our findings align with previous reports showing decreased ATP levels in the habenula, hippocampus, and several other brain regions of mice subjected to CRS or LPS [60]. Compromised ATP levels have been implicated in depressive disorders, and ATP replenishment can effectively modulate depressive-like behaviors in mice. Under physiological conditions, extracellular ATP is mainly released by astrocytes. In these models, ATP treatment successfully reversed depressive behaviors. In our study, we found that miR-129-5p overexpression increased both ATP receptor expression and ATP content, ultimately resulting in improved depression.

Mysm1 serves an important regulator of various epigenetic signaling processes and the innate immune system. Its deficiency results in abnormal hematopoiesis, hyper-inflammation, enhanced viral clearance, and tumor development [61–65]. Additionally, several studies have reported that Mysm1 can interact with the p53 axis during tissue development [62,66]. p53 has been shown to activate the AMPK pathway and suppress the mTOR pathway in glioma cells [67]. Our previous study indicated that Mysm1 was highly expressed in the hippocampus, internal capsule, frontal lobe, and temporal lobe brain sections from patients with severe depression. Similar high expression patterns were observed in the MHb, HIP, or IC of depressed mice [25]. Notably, Mysm1 knockdown in MHb, HIP, or IC alone is sufficient to alleviate depressive-like behaviors in mice. Interestingly, miR-129-5p overexpression also effectively decreased Mysm1 levels in depressive mice.In addition, Chang et al. identified MAPK1 as a direct target of miR-129-5p [58]. Moreover, elevated microRNA-129-5p level ameliorates neuroinflammation and blood-spinal cord barrier damage after ischemia-reperfusion by inhibiting HMGB1 and the TLR3-cytokine pathway.

Research on miR-129-5p in human depression remains limited, with current studies primarily focusing on its expression patterns and potential regulatory mechanisms in mouse models. Nevertheless, several reports have established connections between miR-129-5p and human depression. Studies have revealed decreased expression of miR-129-5p in the extracellular vehicles (EVs) of the brains of patients with depression, suggesting its potential involvement in neurotransmission and synaptic plasticity [38]. This research lays the foundation for further molecular studies exploring the relationship between human depression and miR-129-5p. In future research, it is necessary to verify the expression differences of miR-129-5p in human depression through large-scale clinical samples, as well as its correlation with the severity of depression. Additionally, exploring miR-129-5p’s potential as a biomarker for early diagnosis and assessment of therapeutic effects in depression is also important. The role of miR-129-5p in human depression represents a promising yet understudied field. Future studies will help to elucidate its specific role in depression and may provide new strategies for the treatment of depression.

In summary, this study revealed that overexpression of miR-129-5p alleviated depression-like behavior by increasing ATP production, highlighting the important role of astrocyte miR-129-5p in energy metabolism modulation. Our findings further support that increasing ATP production in astrocytes represents a promising strategy for alleviating depressive disorders. Nevertheless, further investigation is needed to fully elucidate the precise molecular mechanisms by which miR-129-5p modulates the Mysm1/p53/AMPK pathway.

## Supporting information

S1 File Raw images. (DOCX)

## Abbreviations

CRS: Chronic restraint stress
LPS: Lipopolysaccharide
GFAP: Glial fibrillary acidic protein
Iba1: Ionized calcium-binding adaptor molecule 1
IL-6: Interleukin-6
TNF-α: Tumor necrosis factor-alpha
Cx3cr1: C-X3-C motif chemokine receptor 1
Aif-1: Allograft inflammatory factor-1
GLT-1: Glutamate transporter 1
GLAST: Glutamate-aspartate transporter
GS: Glutamine synthetase
