# Supplementary material for: MiR-129-5p alleviates depression and anxiety by increasing astrocyte ATP production partly through targeting deubiquitinase Mysm1
Source: PLoS One. 2025 May 9;20(5):e0322715. doi: 10.1371/journal.pone.0322715 (PMC12064192; doi:10.1371/journal.pone.0322715)
Supplement: S1 File — (DOCX) [file pone.0322715.s001.docx]

**Figure 4I-** GFAP

Sample: Brain tissue

Lane 1. Ctrl

Lane 2. CRS+AAV-CON

Lane 3. CRS+AAV-miR-129-5p


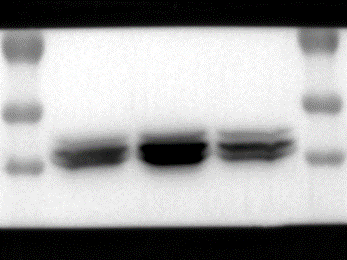

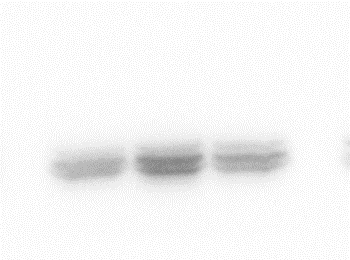

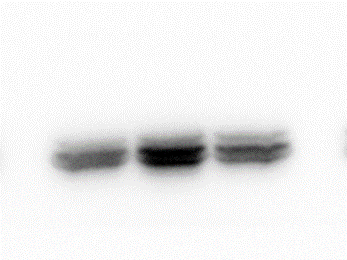


70 kDa

40 kDa

55 kDa

**Figure 4I-** TNFα

Sample: Brain tissue

Lane 1. Ctrl

Lane 2. CRS+AAV-CON

Lane 3. CRS+AAV-miR-129-5p


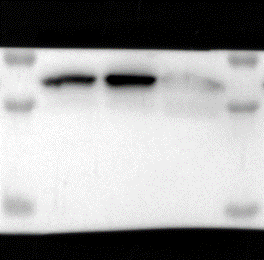

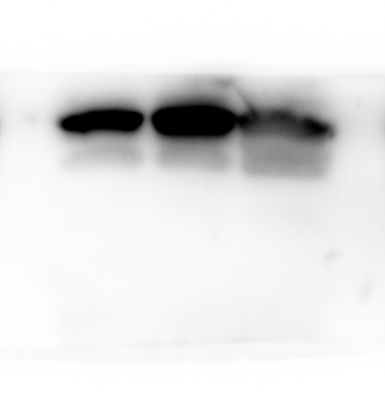

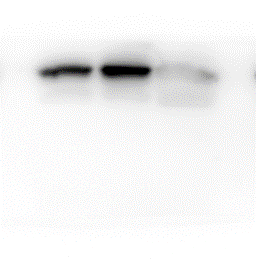


15 kDa

25 kDa

35 kDa

**Figure 4I-** Actin

Sample: Brain tissue

Lane 1. Ctrl

Lane 2. CRS+AAV-CON

Lane 3. CRS+AAV-miR-129-5p


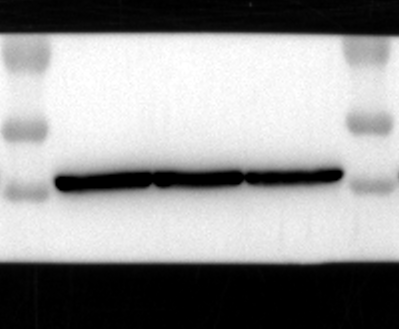

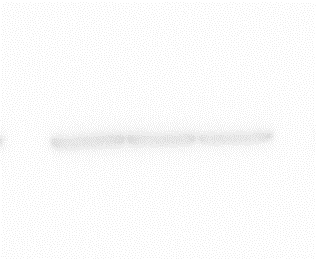

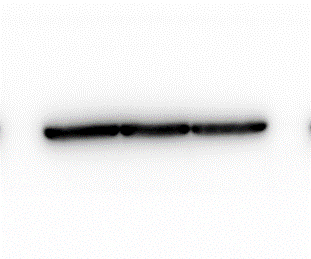


55 kDa

40 kDa

70 kDa

**Figure 6G-** Mysm1

Sample: Brain tissue

Lane 1. Ctrl

Lane 2. CRS+AAV-CON

Lane 3. CRS+AAV-miR-129-5p


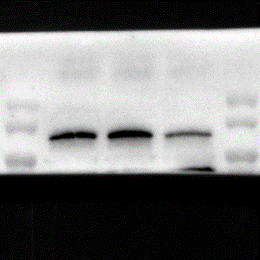

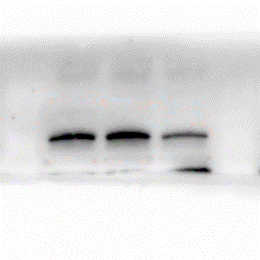

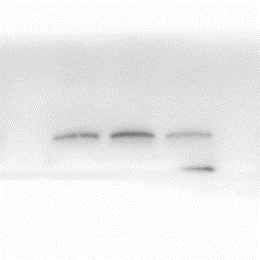


180 kDa

100 kDa

130 kDa

**Figure 6G-** Actin

Sample: Brain tissue

Lane 1. Ctrl

Lane 2. CRS+AAV-CON

Lane 3. CRS+AAV-miR-129-5p


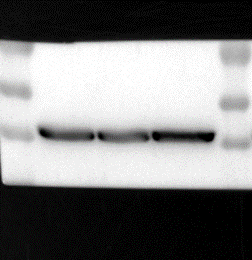

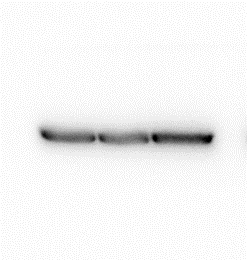

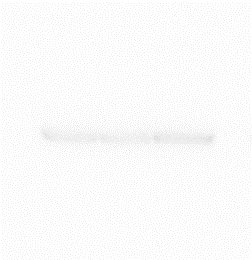


70 kDa

55 kDa

40 kDa

**Figure 6I-** Actin

Sample: Astrocytes

Lane 1. AAV-CON

Lane 2. AAV-miR-129-5p


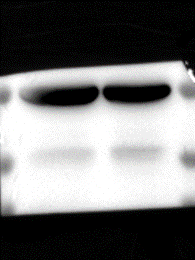

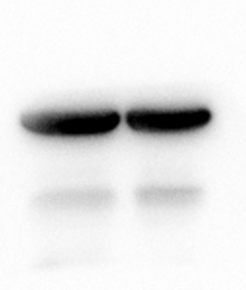

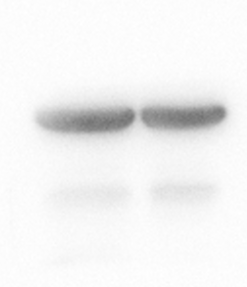


35 kDa

40 kDa

**Figure 6I-** Mysm1

Sample: Astrocytes

Lane 1. AAV-CON

Lane 2. AAV-miR-129-5p


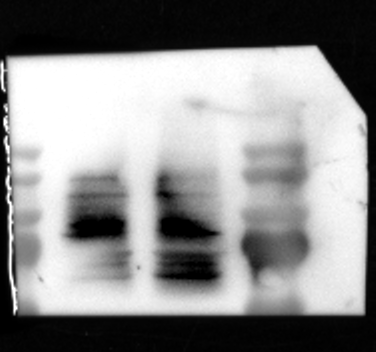

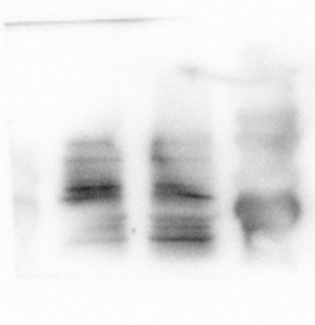

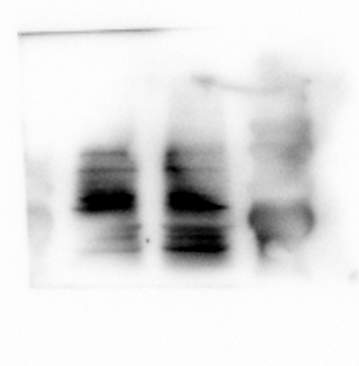


100 kDa

130 kDa

180 kDa

70 kDa

**Figure 6I-** p-p53

Sample: Astrocytes

Lane 1. AAV-CON

Lane 2. AAV-miR-129-5p


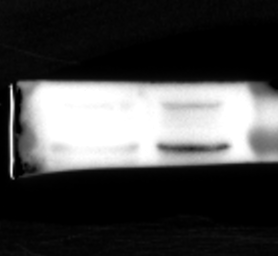

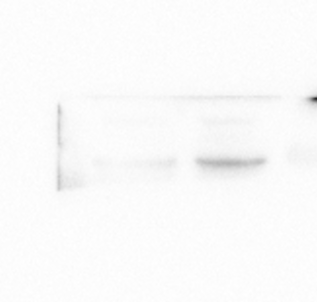

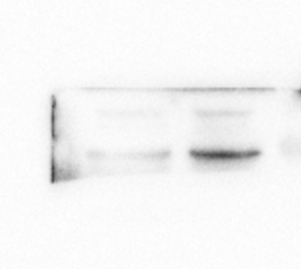


55 kDa

70 kDa

**Figure 6I-** p53

Sample: Astrocytes

Lane 1. AAV-CON

Lane 2. AAV-miR-129-5p


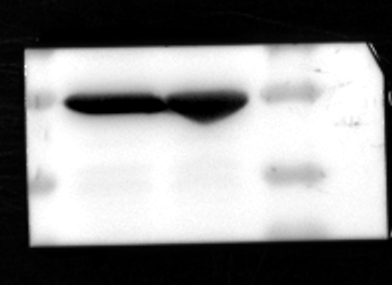

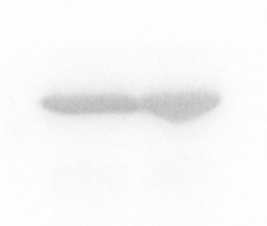

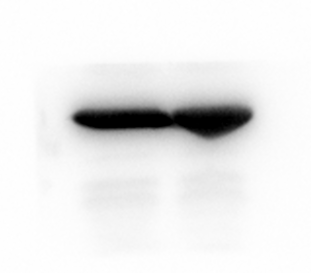


40 kDa

55 kDa

**Figure 6I-** p-AMPK

Sample: Astrocytes

Lane 1. AAV-CON

Lane 2. AAV-miR-129-5p


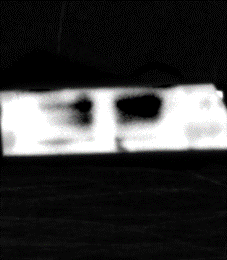

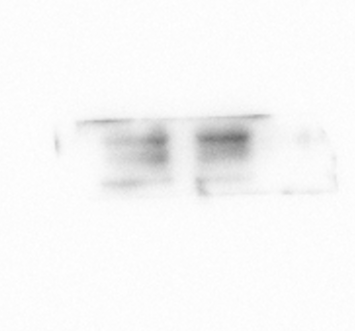

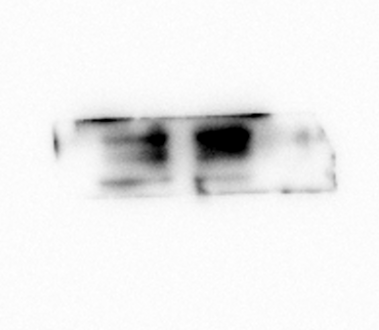


55 kDa

**Figure 6I-** AMPK

Sample: Astrocytes

Lane 1. AAV-CON

Lane 2. AAV-miR-129-5p


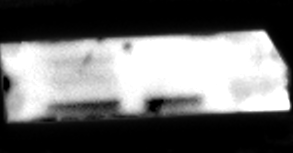

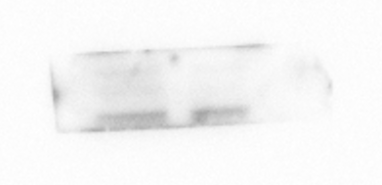

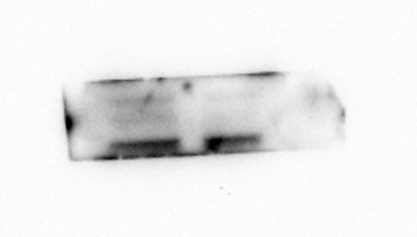


70 kDa

**Figure 6I-** PGC1α

Sample: Astrocytes

Lane 1. AAV-CON

Lane 2. AAV-miR-129-5p


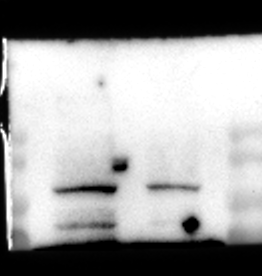

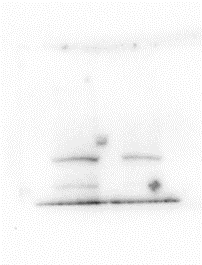

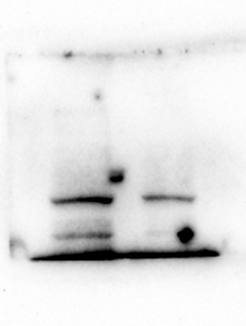


180 kDa

130 kDa

100 kDa

**Figure 6I-** Sirt1

Sample: Astrocytes

Lane 1. AAV-CON

Lane 2. AAV-miR-129-5p


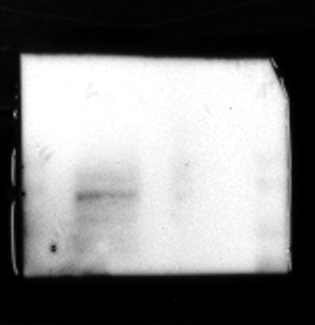

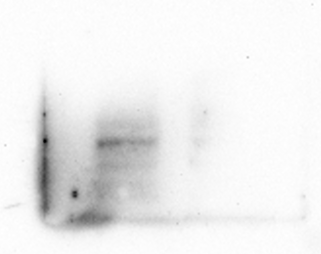

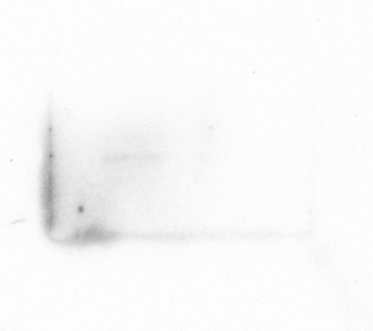


100 kDa

130 kDa
